# Emotion regulation and decision-making in persons with dementia: A scoping review

**DOI:** 10.1177/1471301220971630

**Published:** 2020-11-23

**Authors:** Rotem Perach, Jennifer Rusted, Peter R. Harris, Eleanor Miles, for the DETERMIND team

**Affiliations:** School of Psychology, 1948 University of Sussex, Brighton, UK

**Keywords:** emotional regulation, decision-making, well-being, dementia, review

## Abstract

**Background and Objectives:**

Emotion is integral to decision-making, and emotion regulation is associated with improved well-being in older age. Persons with dementia are likely to experience impairments in emotion regulation processes that can potentially contribute to differential decision-making and well-being outcomes. To promote the development of theoretical models of well-being in dementia, we review the quantitative evidence concerning the associations between emotion regulation and decision-making in dementia.

**Methods:**

Scoping review.

**Results:**

Seven studies of persons with dementia met our criteria. In persons with frontotemporal lobar degeneration, emotion regulation processes that precede the emotional experience were associated with decision-making in a moral (but not uncertainty) context. Independent of type of dementia, evidence concerning the associations between emotion regulation processes that occur after emotion is experienced and decision-making was mixed and drew on different methodologies. No studies relating to the associations between decision-making in dementia and several emotion regulation processes and strategies were found.

**Conclusions:**

In this review, we sought to clarify the concept of everyday decision-making in dementia and map the current state of evidence concerning its associations with emotion regulation. Our findings show that emotion regulation processes are associated with decision-making in dementia, depending on type of decision-making assessment and emotional experience. We outline the gaps in the literature to set a research agenda for promoting our understanding of how emotion regulation processes can shape the various decisions that are made by persons with dementia on a daily basis.

Decision-making pervades the experience of persons with dementia across life contexts (e.g., everyday, care, and financial) (Davis et al., 2017; Groen-van de Ven et al., 2017) and opportunities to continue to engage with decision-making are associated with improved well-being (Haslam et al., 2014; Langer & Rodin, 1976; O'Rourke et al., 2015). Despite the importance of decision-making to outcomes in dementia, little is known on individual resources that could potentially promote it. In this review, we introduce emotion regulation as a key factor that could influence decision-making and well-being in persons with dementia.

Emotion regulation has been defined as “the processes by which individuals influence which emotions they have, when they have them, and how they experience and express these emotions” (Gross, 1998, p. 275). Research has shown that the use and effectiveness of emotion regulation processes and strategies can affect well-being (Charles & Luong, 2013; Ramirez-Ruiz et al., 2019; Wierenga et al., 2017). This may particularly be the case among persons that have limited emotion regulation resources (Urry & Gross, 2010) such as people with limited cognitive resources as happens in dementia (Alzheimer’s Association, 2018).

We propose that emotion regulation could influence outcomes in persons with dementia not only through its effect on well-being, but also through its direct association with decision-making. Emotion has been increasingly recognized as an important element of decision-making (Lerner et al., 2015), and emotion regulation has been associated with decision-making both in theory (Westen & Blagov, 2007) and empirically (Heilman & Miclea, 2015; Higgs et al., 2019; Tamir, 2016). In view of the likely progressive emotional impairments in different dementia subtypes (Kazui et al., 2016) and in order to promote knowledge of the factors that influence decision-making processes and their well-being outcomes, it is important to understand the relationship between decision-making and emotion regulation in dementia.

The present investigation was driven by the desire to promote the development of theoretical models of well-being in dementia that incorporate core aspects of self-regulation (e.g., emotion regulation) (Williams et al., 2018) and decision-making (see Farina et al., 2020 for protocol) and could potentially inform well-being interventions (Berking & Lukas, 2015) in dementia. In this review, we first focus on theory and evidence concerning emotion regulation in older persons. Second, we place research on decision-making in dementia within the context of emotion regulation theory, and third, we complete a scoping review of the quantitative evidence concerning the associations between emotion regulation and everyday decision-making in dementia.

## Emotion regulation processes and strategies

The process model of emotion regulation (Gross, 1998, 2015) is one influential framework for understanding emotion regulation mechanisms and their effects (Allen & Windsor, 2019; Webb et al., 2012). This model outlines five sets of emotion regulation processes that occur before (i.e., antecedent-focused) and after (i.e., response-focused) the emotional experience is generated. *Antecedent-focused processes* include situation selection (actively pursuing or avoiding a situation that one expects to give rise to certain emotions), situation modification (actively altering the external characteristics of a situation to shape its emotional impact), attentional deployment (focusing attention to influence one’s emotional response), and cognitive change (modifying one’s internal situation appraisal to shape its emotional effect). *Response-focused processes* involve a single set of processes (termed response modulation), which involves attempts to influence elements (e.g., experiential, behavioral, and physiological) of the full-blown emotional response (Gross, 1998, 2015). In two meta-analyses of experimental studies, it has been found that emotion regulation is more effective (i.e., more successful in modifying the emotional response) when they target the antecedents of the emotional response than when they target the response itself; for example, in studies that manipulate emotion regulation, cognitive change is generally more effective than response modulation (Brady et al., 2018; Webb et al., 2012).

The effectiveness of emotion regulation depends not only upon which processes are targeted, but also upon which strategy is used to do so. For example, in Webb et al.’s (2012) meta-analysis of 190 experimental studies, within cognitive change strategies perspective-taking was more effective in modifying emotional responses than was reappraisal. Thus, the use of different emotion regulation strategies can lead to different outcomes. Below, we describe how these emotion regulation processes and strategies differ in older age, before considering their associations with decision-making in dementia.

## Emotion regulation in older age

Current theory suggests that age is one factor that may influence the use and effectiveness of emotion regulation strategies (Carstensen et al., 1999; Charles & Luong, 2013; Urry & Gross, 2010). From a socioemotional selectivity theory perspective, with age, people are increasingly motivated to pursue meaningful emotional goals and to avoid potentially negative emotional experiences (Carstensen et al., 1999). Accordingly, older (vs. younger) persons are more likely to rely on antecedent-focused emotion regulation strategies that enable construction of a structured social world (e.g., selecting for likely pleasant social interactions) (Carstensen et al., 1999). Others have suggested that among older persons, emotion regulation strategies may be shaped by one’s internal (e.g., acquired knowledge on the effects of emotional expression) and external (e.g., having a social network that supports positive interchanges) resources (Urry & Gross, 2010). Thus, various theories have supported age-related differences in the use and effectiveness of emotion regulation strategies.

Empirical evidence suggests that these differences are not necessarily straightforward and may depend on various moderators (Allen & Windsor, 2019; Livingstone & Isaacowitz, 2016). In a systematic review examining age differences in the use of emotion regulation strategies, evidence supported the notion that older persons prefer using situation selection and attentional deployment strategies, depending on context and individual characteristics (Allen & Windsor, 2019). For example, in order to avoid experiencing negative emotions, older persons may prefer to sidestep interpersonal conflicts (Charles et al., 2009). In terms of effectiveness, no age-related differences were found in instructed attentional deployment, cognitive change, or response modulation strategies in a meta-analysis of 11 experimental studies (Brady et al., 2018). Likewise, age did not moderate effect sizes of the effectiveness of emotion regulation strategies in another meta-analysis (Webb et al., 2012). However, caution is warranted when interpreting the latter findings in view of the under recruitment of samples of older persons (Webb et al., 2012). Thus, there is some evidence that older persons regulate their emotions differently but no less effectively.

One factor that may help to explain these findings is age-related differences in emotion regulation resources, that is, internal or external factors that enable the use of a particular emotion regulation process or strategy (Urry & Gross, 2010). For example, the capacity to develop and carry out plans could affect a person’s ability to successfully implement situation selection or modification. Similarly, perspective-taking, memory, and cognitive control could influence cognitive change, and behaviors that can affect emotional experience could affect response modulation (Urry & Gross, 2010). It follows that the populations in which such resources become increasingly limited (e.g., persons with dementia) are likely to have characteristics that may contribute to differences in the use and effectiveness of emotion regulation, and thus to differential decision-making and well-being outcomes. Indeed, effective emotion regulation is associated with improved well-being in populations with chronic diseases (Wierenga et al., 2017) and in older persons (Charles & Luong, 2013).

## Emotion regulation and decision-making in dementia

In this section, we consider evidence on how emotion regulation processes are affected in persons with dementia and the interconnections between emotion regulation and decision-making in this population. Dementia is an umbrella term for a number of neurodegenerative diseases that typically affect older persons and are each associated with a different set of symptoms. All dementia subtypes are characterized by a progressive cognitive decline severe enough to interfere with daily living and independent functioning (Alzheimer’s Association, 2018; National Health Service, 2017). Persons with different dementia subtypes are likely to experience impairments in both antecedent- and response-focused emotion regulation, as detailed next.

### Emotion regulation in dementia

#### Antecedent-focused

Considering the limited cognitive resources in dementia, it is feasible that persons with dementia of any type can have impaired antecedent-focused emotion regulation processes. Indeed, persons with behavioral variant of frontotemporal dementia (bvFTD), a subtype of frontotemporal lobar degeneration (FTLD), can experience difficulties in emotion recognition and representational abilities such as affective theory of mind abilities (i.e., representation/recognition of the affective mental states of other people to understand or predict their behavior; Enrici et al., 2015). In a meta-analysis, significant impairments in theory of mind abilities were found in persons with bvFTD in comparison to healthy controls (Bora et al., 2015). In addition, the findings of a review on studies of emotion regulation in persons with dementia of Alzheimer’s type (DAT) show that, as the disease progresses, facial emotion recognition is overall impaired (Torres Mendonça De Melo Fádel et al., 2019). In a meta-analysis of persons with DAT, theory of mind abilities were impaired in comparison to healthy controls (Bora et al., 2015). Finally, a review showed evidence in support of the notion that among persons with DAT (in different stages), some (cognitively taxing) emotion regulation abilities may be impaired whereas other emotion regulation abilities can remain intact despite cognitive decline (Fischer et al., 2019). Overall, antecedent-focused emotion regulation processes can be impaired in dementia including due to limited cognitive resources and/or emotion recognition and representation difficulties.

#### Response-focused

Several dementia subtypes (i.e., DAT, bvFTD, Parkinson’s disease dementia (PDD), dementia with Lewy bodies (DLB), and vascular dementia) are associated with changes in emotional experience (National Health Service, 2017), and these will affect response-focused emotion regulation (Brans et al., 2013; Gross, 2015). Symptoms of bvFLD, for example, can include apathy, and loss of empathy and sympathy (Rascovsky et al., 2011). In DAT, symptoms can include apathy, loss of empathy, depressive symptoms, and emotion regulation difficulties (Alzheimer’s Association, 2018). Depressive symptoms, anxiety, and apathy can be experienced by persons with PDD (Emre et al., 2007) and persons with DLB (Capouch et al., 2018). In the same vein, according to the International Classification of Diseases, 10th Revision (ICD-10), depressive symptoms and emotional liability are among the supportive features of vascular dementia (Wiederkehr et al., 2008).

In sum, both emotion regulation resources and emotional experiences are affected in persons with DAT, bvFLD, PDD, DLB, and vascular dementia and are therefore likely to influence the characteristics (e.g., use and effectiveness) and outcomes (e.g., decision-making and well-being) of both antecedent- and response-focused emotion regulation strategies and processes in these populations.

### Everyday decision-making in dementia

Persons with dementia make decisions across many areas of their lives including everyday, social, care planning, and financial contexts (Davis et al., 2017; Groen-van de Ven et al., 2017). Given our focus on self-regulation processes (Farina et al., 2020), this review focused on daily decisions that are made by the person with dementia and relate to one’s desires including regarding personal (e.g., eating, dressing) and social activities (termed *everyday decision-making*; Davis et al., 2017). Everyday decision-making in persons with dementia can be evaluated by decision-making tasks (De Siqueira et al., 2017), self-report scales, and interview-based assessments (Davis et al., 2017). Across assessments, little is known concerning the association between emotion regulation and everyday decision-making in dementia.

Decision-making tasks have been widely used to understand everyday decision-making processes and impairments in various populations (Aram et al., 2019; Jacus et al., 2018). Such tasks include two main types, namely, decision-making under uncertainty (in which premises, outcomes, and feedback are initially unknown) and decision-making under risk (in which the potential outcomes and outcome probability of different options are known or can be inferred using available information) (Ryterska et al., 2013) (various decision-making tasks draw on the above principles; see Supplementary Appendix 1 for descriptions of selected tasks). Reviews that have evaluated performance on decision-making tasks have shown that (based on few studies) persons with probable DAT, FTLD, or PDD can show impairments in both decision-making under uncertainty and under risk (De Siqueira et al., 2017; Gleichgerrcht et al., 2010). However, the associations between emotion regulation and performance on decision-making under uncertainty/risk tasks in these populations have not been examined as a whole.

An additional type of a decision-making task concerns decisions that are made in moral contexts (henceforth, moral decision-making). These are typically assessed by asking participants to make a decision relating to a vignette presenting a moral dilemma (e.g., the trolley/footbridge dilemma; Thomson, 1976). Everyday moral behavior is progressively affected in persons with FTLD (Neary et al., 1998). Considering that impaired moral emotion processing has been reported among persons with bvFTD in comparison to age- and disease duration-matched persons with DAT and healthy, age-matched controls (Teichmann et al., 2019), it is therefore feasible that everyday moral decisions are associated with emotion regulation in bvFTD. Indeed, Mendez and colleagues have reported that emotion-based moral decision-making is affected in persons with FTLD (Mendez et al., 2005; Mendez & Shapira, 2009). Overall, these findings highlight the need to examine in more detail the evidence concerning the association between emotion regulation and moral decision-making in dementia.

### The current review

Emotion regulation has been associated with decision-making, and both constructs have been associated with well-being. Due to the progressive cognitive decline in dementia, persons with dementia have limited emotion regulation resources and therefore potentially impaired antecedent-focused emotion regulation (Urry & Gross, 2010). In addition, people with different dementia subtypes can experience changes in emotional experience that influence response-focused emotion regulation (Brans et al., 2013; Gross, 2015). Across the time course of the developing dementia, persons with dementia will differ in their decision-making capacity (Mitoku & Shimanouchi, 2014) and individuals’ emotion regulation characteristics may contribute to these differences (Urry & Gross, 2010). Understanding the associations between emotion regulation impairments and decision-making in dementia could therefore inform the development of well-being models in dementia. In addition, given the modifiable nature of emotion regulation capacities (Berking & Lukas, 2015), a deeper understanding of its influence on decision-making and consequent well-being can potentially promote the development of self-regulation-based interventions (Farina et al., 2020) and enhance existing interventions that incorporate emotion regulation and/or decision-making components/evaluations (e.g., see Oyebode and Parveen, 2019). Consistent with the above aims, this scoping review examines quantitative evidence concerning the associations between emotion regulation and decision-making in dementia.

## Methods

### Search methodology

#### Strategy selection

A scoping review methodology is appropriate for researching the available types of evidence and their corresponding research methods in a particular field, clarifying key concepts and identifying their related factors, and analyzing knowledge gaps (Munn et al., 2018). As detailed in the Introduction, emotion regulation and decision-making in dementia are complex concepts relating to heterogenous bodies of the literature. In the current study, we aimed to investigate the various constellations of associations between different characteristics of emotion regulation and of decision-making in dementia, thereby enabling the identification of knowledge gaps relating to the development of theory concerning self-regulation and decision-making in dementia (Farina et al., 2020). Accordingly, and based on the guidance provided by Munn et al. (2018), scoping review methodology was employed.

### Search strategy

We searched the quantitative literature by identifying 11 reviews (published by December, 2019; five of which were published between 2015 and 2019) that included information on studies of persons with dementia that used a decision-making task (Christidi et al., 2018; De Siqueira et al., 2017; Elamin et al., 2012; Gleichgerrcht et al., 2010; Perry & Kramer, 2015), an emotion regulation task (Fischer et al., 2019), and decision-making measures (Capucho & Brucki, 2011; Davis et al., 2017; Karlawish, 2008; Lai & Karlawish, 2007; Law et al., 2011). Next, we manually examined their references. We identified further articles using the “cited by” function on Google Scholar for reviews and included studies and then manually examined their references. Finally, we located records that were familiar to the authors.

### Inclusion and exclusion criteria

*Inclusion criteria* were 1. participants (full sample or a distinct group) had (any type of) dementia based on a diagnosis by a clinical neurologist or were described as meeting published clinical diagnostic criteria; 2. peer-reviewed journal article that reported new quantitative data in English on the association between everyday decision-making and emotion regulation in persons with dementia; 3. we operationalized everyday decision-making as both tasks classified as assessing everyday decision-making (i.e., under uncertainty, under risk, and moral) and measures labeled as decision-making assessments. Decision-making measures that did not assess daily decisions made by the person with dementia (e.g., clinician-assessed decision-making capacity (Karlawish, 2008), legal competence (Moye et al., 2011)), primarily evaluated everyday cognitive competence (e.g., memory and problem-solving), or used hypothetical scenarios that may potentially limit some insight into real-life decision-making (Lai & Karlawish, 2007) were not included (see Supplementary Appendix 1 for decision-making tasks’ terminology, and decision-making measures that met our criteria); 4. in line with Gross (1998), we operationalized emotion regulation as: a) assessments of antecedent-focused emotional processes. These included self-report measures of emotion recognition and representation (e.g., affective theory of mind abilities) that reflect cognitive construction of an emotional situation (Enrici et al., 2015), physiological measures of skin conductance response (an index of emotional processing; Braithwaite et al., 2013), and experimental tasks that assess antecedent-focused emotional processes (Webb et al., 2012); b) assessments of response-focused emotional processes. For example, self-report measures that assess emotional experience as a single construct (e.g., anxiety) or as one factor of a compound construct (e.g., health-related quality of life) and experimental tasks that assess response-focused emotional processes (Webb et al., 2012); and c) tasks/measures labeled as measuring emotion regulation. Neuroanatomical assessments of emotion-related structures were incompatible with our focus (Farina et al., 2020) and therefore excluded; 5. while decision-making in dementia may involve others, our focus was on the person with dementia’s perspective on individual factors that may influence everyday decision-making (Farina et al., 2020). Therefore, we only included studies in which the person with dementia was the source of evidence (De Boer et al., 2007); and 6. to rule out intervention effects on the associations under study, in intervention studies, we only considered preintervention data for inclusion. *Exclusion criteria* were 1. emotion was not assessed independently of decision-making (e.g., moral dilemmas were labeled as “emotional”; Mendez & Shapira, 2009); and 2. qualitative studies were excluded to promote the development of interventions that are based on quantitative evidence. Nonetheless, the investigation was informed by qualitative research (e.g., DeBoer et al., 2007; O’Rourke et al., 2015). See Supplementary Appendix 2 for a list of excluded quantitative studies.

### Quality assessment

In line with scoping review guidelines provided by Arksey and O’Malley (2005), we exclude quality assessment of individual studies. In the Discussion, we comment on the methodological characteristics and diversity of the overall evidence.

## Results

**Figure 1.**
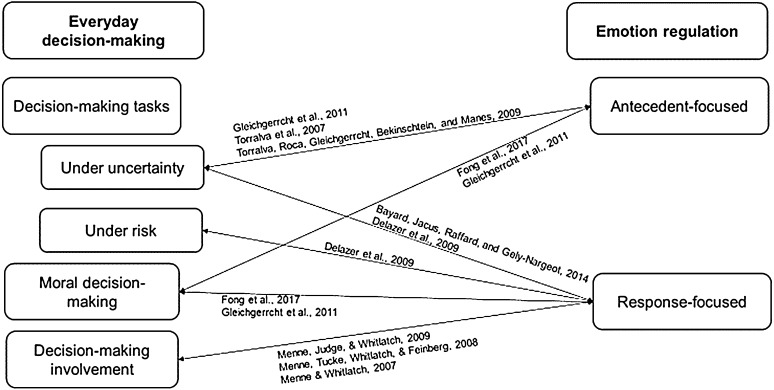
Visual representation of evidence concerning the associations between emotion regulation processes and types of everyday decision-making in dementia.

Seven studies (reported in nine studies) met our criteria. The characteristics of studies that used decision-making tasks are presented in Table 1 and of studies that used a measure of decision-making in Table 2. Evidence concerning the associations between emotion regulation processes and types of everyday decision-making in dementia is mapped in Figure 1. Findings are presented below by dementia subtype and emotion regulation processes.

**Table 1.** Characteristics of studies that used decision-making tasks (*N* = 6).

	Participants by group	Age *M* (*SD*)	Gender (% female)	MMSE *M* (*SD*)	Location	Decision-making task	Emotion regulation		Findings in dementia group(s)
							Antecedent-focused	Response-focused	
Study									
Bayard et al. (2014)	G_1_ = 20 persons with DAT G_2_ = 20 persons with amnestic MCI CG = 20 cognitively intact persons G_1_ = 20 persons with idiopathic PD without dementia G_2_ = 19 persons with PDD CG = 20 cognitively intact persons	G_1_ = 80.9 (5.4) G_2_ = 78.25 (6.9) CG = 73.5 (6.7)^a^ G_1_ = 68.5 (5.9) G_2_ = 72.2 (5.1) CG = 71.3 (3.5)	G_1_ = 60 G_2_ = 55 CG = 55 G_1_ = 25 G_2_ = 57.89 CG = 85	G_1_ = 24.8 (2.3)^b^ G_2_ = 27.15 (2.0)^c^ CG = 28.5 (.9) G_1_ = 27.8 (1.9)^d^ G_2_ = 24.7 (3.0) CG = 29.8 (.4)^e^	France Austria	Iowa gambling task Iowa gambling task and probability-associated gambling task		Lille apathy rating scale HADS-anxiety and HADS-depression	There was no significant group by Iowa gambling task profile (advantageous vs. disadvantageous) interaction on total apathy or its sub-dimensions. In an analysis that pooled all groups, participants with advantageous (vs. disadvantageous) Iowa gambling task profile showed significantly less apathy on the lille apathy rating scale action initiation dimension (but not on the other three dimensions). This was found for both the Iowa gambling task total score, *p* = .04, Cohen’s *d* = .59, and for Iowa gambling task score based on blocks 3-5, *p* = .005, Cohen’s *d* = .79. No significant correlation was found between either decision-making task and either anxiety or depressive symptoms.
Delazer et al. (2009)									
Fong et al. (2017) ^f^	G_1_ = 10 persons with bvFTD G_2_ = 11 persons with probable DAT CG = 9 cognitively intact persons	G_1_ = 62.40 (11.51) G_2_ = 61.36 (5.70) CG = 53.88 (9.51)	G_1_ = 63.64 G_2_ = 75 CG = 69.23	G_1_ = 27.40 (2.41) G_2_ = 25.30 (3.30) CG = 29.50 (.9)^g^	US	Moral decision-making (trolley switch and footbridge)	Skin conductance response	Feelings about decision	Across the two moral dilemmas, there was a significant effect of group by (positive or negative) emotion, *p* < .05, *p* < .01, resp. G_1_ participants had significantly more positive emotions compared to both G_2_, *p* = .01, and CG, *p* = .01, participants. Also, G_1_ participants had significantly fewer negative emotions compared to CG, *p* < .001. Differences in negative emotions between G2 and CG participants were nonsignificant, *p* = .053. There was a significant group by (mental processing of) moral dilemma interaction on skin conductance responses so that persons with bvFTD showed an opposite pattern of autonomic arousal in comparison to G_2_ and CG participants on moral decision-making dilemmas. There were no significant skin conductance response differences between G_2_ and CG participants. In a regression analysis, the interaction of G_1_ (but not G_2_) by footbridge dilemma significantly predicted decreased skin conductance responses.
Gleichgerrcht et al. (2011)	G_1_ = 9 persons with early/mild bvFTD (decision: Push) G_2_ = 13 persons with early/mild bv FTD (decision: Not push)	G_1_ = 71.2 (6.80) G_2_ = 71.4 (5.46)	G_1_ = 66.67 G_2_ = 38.46	G_1_ = 23.2 (4.32) G_2_ = 22.7 (5.96)	Argentina	Iowa gambling task and moral decision-making (footbridge)	Faux Pas test and reading the mind in the eyes task	Interpersonal reactivity inventory	Participants who made the decision to push (vs. not push) man onto train tracks on footbridge dilemma showed significantly lower reading the mind in the eyes scores, U = 9.00, *p* = .02, and did not differ on Faux pas scores or (total or subscale) empathy scores. There was no significant correlation between reading the mind in the eyes and the Iowa gambling task (block 5) scores within both groups.
Torralva et al. (2007)	G_1_ = 20 persons with early/mild fvFTD CG = 10 cognitively intact persons	G_1_ = 67.2 (8.1) CG = 63.5 (5.8)	G_1_ = 45 CG = 60	G_1_ = 27.9 (1.6) CG = 29.5 (.8)^h^	Argentina	Iowa gambling task	Faux Pas test and reading the mind in the eyes task		There were no significant correlations between decision-making and either theory of mind measure.
Torralva et al. (2009) ^i^	G_1_ = 16 persons with early/mild bvFTD and higher ACE scores G_2_ = 19 persons with early/mild bvFTD and lower ACE scores CG = 14 cognitively intact persons	G_1_ = 65.0 (7.4) G_2_ = 69.1 (5.7) CG = 65.5 (6.5)	G_1_ = 56.25 G_2_ = 52.63 CG = 50	G_1_ = 28.2 (1.9) G_2_ = 25.7 (3.2)^j^ CG = 29.2 (1.0)	Argentina	Iowa gambling task	Faux Pas test and reading the mind in the eyes task		There were no significant correlations between decision-making and either theory of mind measure, independent of cognitive status scores.

*Note.* This tables includes only measures/tasks that were examined in connection to the relationship between decisions-making and emotion-related measures. Level of significance is *p* < .05. Significant group differences on age or MMSE scores are annotated in the corresponding column in the table where applicable. ACE: Addenbrooke’s Cognitive Examination; CG: control group; DAT: dementia of Alzheimer’s type; HADS: Hospital Anxiety and Depression Scale; bvFTD: behavioral variant of frontotemporal dementia; fvFTD: frontal variant of frontotemporal dementia; G: group; MCI: mild cognitive impairment; MMSE: mini-mental state examination; PD: Parkinson’s disease; PDD: Parkinson’s disease dementia.

^a^Significantly younger in comparison to G_1_, *p* < .001 and G_2_, *p* < .05.

^b^Significantly lower scores in comparison to G_2_, *p* < .01 and G_3_, *p* < .05 with age and education as covariates.

^c^Significantly lower scores in comparison to G_3_, *p* < .05 with age and education as covariates.

^d^Significantly higher scores in comparison to G_2_.

^e^Significantly higher scores in comparison to G_1_ and G_2_.

^f^Percentage of female participants per group is based on rates reported in the Results section.

^g^Significantly higher scores in comparison to G_2_, *p* = .001.

^h^Significantly higher scores in comparison to G_1_, *p* < .01.

^i^In the analyses reported in this study, bvFTD participants were allocated into grouped based on their ACE score; G_1_ = ACE > 86; G_2_ = ACE ≤ 86.

^j^Significantly lower scores in comparison to G_1_ and CG, *p* < .001.

**Table 2.** Characteristics of studies that used a measure of decision-making that met our criteria (*n* = 1).

	Participants^a^	Age *M* (*SD*)	Gender (% female)	Location	MMSE *M* (*SD*)	Decision-making scale	Emotion regulation	Findings
							Response-focused	
Study								
Menne et al. (2009)	211 persons with dementia	76.07 (8.95)	51	US	22.01 (4.67)	Decision-making involvement	QoL-AD	Lower decision-making involvement scores were a significant positive predictor of quality of life, β = .23, *p* < .01.
Menne et al. (2008)	217 family caregiver–person with dementia dyads	76 (9.23)	50	US	22 (4.66)	Decision-making involvement	CES-D, Revised Memory and Behavior Problem Checklist, QoL-AD, and dyadic relationship strain scale	Lower decision-making involvement scores were negatively associated with depression symptoms as assessed by the Revised Memory and Behavior Problem Checklist, *r* = −.23, *p* < .01, and positively associated with quality of life, *r* = .30, *p* < .01. Decision-making involvement scores were not significantly associated with depression as assessed by the CES-D, positive relationship strain, or negative relationship strain.
Menne and Whitlatch (2007)	215 family caregiver–person with dementia dyads	75.89 (9.26)	50	US	21.98 (4.65)	Decision-making involvement	Dyadic relationship strain scale, burden to family	Perceptions of avoiding being burden to family were positively associated with decision-making involvement levels, *r* = .31, *p* < .01, but did not significantly predict decision-making involvement in a multivariate analysis. No associations between decision-making involvement levels and either negative dyadic strain or positive dyadic interaction were found.

*Note.* This table includes three papers that present different analyses of a single data source. Only information on measures in which the person with dementia is the source of evidence is presented. CES-D: center for epidemiological studies depression scale; QoL-AD: quality of life-Alzheimer disease scale.

^a^In all studies in the table, sample included persons with a confirmed diagnosis (or symptoms) of a memory impairing condition (e.g., DAT, vascular dementia, and nonspecific or other dementia) or an MMSE score indicating mild–moderate cognitive impairment.

### Persons with bvFTD

#### Antecedent-focused

Three studies reported on the association between decision-making and antecedent-focused processes in persons with bvFTD (Fong et al., 2017; Gleichgerrcht et al., 2011; Torralva et al., 2009). In one study, significant negative associations were found between decision-making on a moral dilemma and reading the mind in the eyes task scores among persons with early/mild bvFTD (Gleichgerrcht et al., 2011). Specifically, persons with early/mild bvFTD who made the decision to push (vs. not push) man onto train tracks to save five workmen on the footbridge dilemma showed significantly lower reading the mind in the eyes scores. In the same study, reading the mind in the eyes scores were not associated with decision-making under uncertainty in either group (push, not push), and the two groups did not significantly differ on Faux Pas test scores (Gleichgerrcht et al., 2011).

Another study reported the associations between moral decision-making and skin conductance response (Fong et al., 2017). In this study, three groups of participants (i.e., bvFTD, age-matched DAT, and cognitively intact controls) completed one of two moral dilemmas (i.e., footbridge and trolley) and were asked about their feelings about the decision they made. Skin conductance response was measured before and during the completion of the moral dilemma. A significant interaction was found such that persons with bvFTD showed an opposite pattern of autonomic arousal in comparison to persons with DAT and to controls when mentally processing a moral dilemma. There were no significant skin conductance response differences between DAT and control participants. The interaction of bvFTD (but not DAT) by (mental processing of) footbridge dilemma significantly predicted decreased autonomic arousal. In other words, emotional arousal as indicated by autonomic activity was decreased among persons with bvFTD faced with a moral dilemma that involved causing direct personal harm (Fong et al., 2017). Finally, in another study, no significant correlations were found between Iowa gambling task scores and reading the mind in the eyes or Faux Pas scores among persons with early/mild bvFTD, independent of cognitive status scores (Torralva et al., 2009).

#### Response-focused

In the aforementioned study by Gleichgerrcht et al. (2011), the two groups (push vs. not push) did not significantly differ on empathy scores. In the above study by Fong et al. (2017), across the two moral dilemmas, there was a significant effect of group on (positive and negative) emotion about their decision. Specifically, persons with bvFTD expressed significantly more positive emotions (in comparison to persons with DAT and to controls) and fewer negative emotions (in comparison to controls). Differences in negative emotions between DAT and control participants were nonsignificant.

### Persons with frontal variant of fronto-temporal dementia

#### Antecedent-focused

In a study of persons with early/mild frontal variant of fronto-temporal dementia (fvFTD), decision-making under uncertainty was not associated with scores on the reading the mind in the eyes task and Faux Pas test (Torralva et al., 2007).

### Persons with PDD

#### Response-focused

In one study, among persons with PDD, neither depressive symptoms nor anxiety were associated with decision-making under uncertainty or under risk (Delazer et al., 2009).

### Persons with DAT

#### Response-focused

In one study, apathy and decision-making on the Iowa gambling task were assessed in three groups of participants (i.e., DAT, amnestic mild cognitive impairment, and cognitively intact controls) (Bayard et al., 2014). No significant group by Iowa gambling task profile (advantageous vs. disadvantageous) interaction on total apathy or its subdimensions was found. In an analysis that pooled all groups, participants with advantageous (vs. disadvantageous) Iowa gambling task profile showed significantly less apathy on the apathy action initiation dimension (i.e., low initiative and everyday productivity), but not on the other three dimensions (Bayard et al., 2014). Additional findings on persons with DAT obtained by Fong et al. (2017) were described above, reporting no difference from controls in autonomic arousal on moral dilemma tasks.

### Mixed dementia populations

#### Response-focused

One study (whose data are analyzed in three studies) reported on the associations between decision-making involvement and response-focused emotion regulation (Menne & Whitlatch, 2007; Menne et al., 2008, 2009) (Table 2). The population under study included persons with a confirmed diagnosis (or symptoms) of a memory impairing condition (e.g., DAT, vascular dementia, and nonspecific or other dementia) or an MMSE score indicating mild–moderate cognitive impairment (Menne & Whitlatch, 2007).

Decision-making involvement levels were positively associated with perceptions of avoiding being a burden to the family in one study (Menne & Whitlatch, 2007). Also, decision-making involvement scores were associated with depressive symptoms as assessed by the Revised Memory and Behavior Problem Checklist in one study (Menne et al., 2008) but not as assessed by the Center for Epidemiological Studies Depression Scale in two studies (Menne & Whitlatch, 2007; Menne et al., 2008). Quality of life in DAT was positively associated with decision-making involvement scores in one study (Menne et al., 2008) and predicted by decision-making involvement scores in a multivariate analysis in another (Menne et al., 2009). Finally, no associations between decision-making involvement levels and either negative dyadic strain or positive dyadic interaction were found in two studies (Menne & Whitlatch, 2007; Menne et al., 2008). Overall, the above evidence shows that in analyses based on a single data source of US persons with dementia, decision-making involvement was associated with emotion-related constructs including perceptions of burden to family and quality of life and showed inconclusive associations with depressive symptoms (Table 2).

## Discussion

This review examined the associations between everyday decision-making and emotion regulation in dementia. We found mixed evidence concerning the associations between decision-making and both antecedent-focused and response-focused emotion regulations in dementia in seven studies of persons with bvFTD, fvFTD, DAT, and PDD. Poorer antecedent-focused emotion regulation in persons with bvFTD/fvFTD was associated with contemplating the decision to or deciding to cause direct personal harm in two studies. However, antecedent-focused emotion regulation was not associated with decision-making under uncertainty in three studies of persons with bvFTD/fvFTD. Evidence concerning the associations between response-focused emotion regulation processes and decision-making was mixed and derived from studies using a range of methodologies and different dementia populations. Overall, the findings of this scoping review show that processes of emotion regulation are associated with everyday decision-making in dementia, depending on type of everyday decision-making and emotional experience.

Our mapping of the evidence to date revealed important gaps in the dementia literature. We found no studies relating to the associations between decision-making in dementia and several of the antecedent-focused emotion regulation processes that, according to the current theory, can potentially contribute to decision-making in dementia. Also, the studies identified that address response-focused emotion regulation in association with decision-making focused exclusively on investigating emotional experience, whereas the use of response-focused emotion regulation strategies was not investigated. Taken together, the current findings represent a crucial first step toward understanding how emotion regulation processes can shape everyday decision-making in dementia and identify understudied aspects of this association. Below, we discuss the evidence concerning the associations between everyday decision-making and emotion regulation and outline knowledge gaps with respect to the different emotion regulation processes.

### Antecedent-focused emotion regulation and decision-making in dementia

Evidence concerning the association between antecedent-focused emotion regulation and decision-making in dementia was obtained in a small number of studies (i.e., four) of persons with bvFTD/fvFTD, of which three had a cognitively intact control group (Table 1). Concerning antecedent-focused processes, this review showed that in two studies, moral decision-making was associated with lower emotion recognition or arousal. Specifically, making the decision to push (vs. not push) a man onto train tracks to save five workmen on the footbridge dilemma was associated with lower emotion and mental state recognition (Gleichgerrcht et al., 2011) and contemplating the footbridge dilemma was associated with decreased emotional arousal (Fong et al., 2017). This finding is in line with evidence that moral emotion processing is impaired in bvFTD (Teichmann et al., 2019) and that emotion may influence moral decision-making in FTLD (Mendez & Shapira, 2009; Mendez et al., 2005). In addition, in one study, moral decision-making was not associated with the comprehension of inappropriate expressions in social situations (Gleichgerrcht et al., 2011). Overall, based on the above three studies, evidence shows that contemplating or making the decision to cause direct personal harm was associated with poorer antecedent-focused emotion regulation (i.e., emotion recognition and arousal, but not representation) in persons with bvFTD/fvFTD. Nonetheless, further research on the association between these indices of antecedent-focused emotion regulation and moral decision-making in FTLD is needed before strong conclusions can be made. In addition, no associations were found between decision-making under uncertainty and emotion recognition (Gleichgerrcht et al., 2011; Torralva et al., 2007, 2009) and understanding social exchanges (Torralva et al., 2007, 2009) in persons with bvFTD/fvFTD. Taken together, the findings of this review show that processes of antecedent-focused emotion regulation are associated with decision-making in moral (but not uncertainty) contexts in FTLD.

In the four studies identified, evidence relating to antecedent-focused processes was limited to measures of the cognitive construction of an emotional event and emotional arousal. We did not find studies on the evaluation of various other antecedent-focused processes and strategies that may contribute to decision-making in dementia, such as the use of situation selection strategies (Carstensen et al., 1999) or cognitive change strategies (Urry & Gross, 2010). Accordingly, future research on the relationships between different antecedent-focused emotion regulation processes and everyday decision-making in persons with different dementia subtypes is needed. In addition, whereas decision-making under uncertainty and moral decision-making were assessed, we found no studies of decision-making under risk (Figure 1). Considering that decision-making under risk may be impaired in different dementia subtypes (e.g., DAT, fvFTD) (De Siqueira et al., 2017; Gleichgerrcht et al., 2010) and is potentially associated with individuals’ emotion regulation resources and how these contribute to antecedent-focused processes (Urry & Gross, 2010), future research on the relationships between decision-making under risk and antecedent-focused processes in dementia is needed.

### Response-focused emotion regulation and decision-making in dementia

The association between response-focused emotion regulation and decision-making in dementia was examined in five studies of persons with bvFTD, PDD, DAT, and mixed dementia populations, of which three had a cognitively intact control group (Table 1, Table 2). Concerning response-focused processes, evidence on the associations between response-focused emotion regulation processes and decision-making is overall mixed and included diverse methodologies (e.g., emotion regulation/decision-making assessments) and populations under study. Specifically, significant associations were found between decision-making under uncertainty and apathy (Bayard et al., 2014), between emotions related to completing a moral dilemma and dementia subtype (Fong et al., 2017), and between decision-making involvement and burden to family (Menne & Whitlatch, 2007), quality of life in DAT (Menne et al., 2008, 2009), and depressive symptoms (Menne et al., 2008). Null associations were found between decision-making assessments and emotional experiences (Bayard et al., 2014; Delazer et al., 2009; Gleichgerrcht et al., 2011; Menne & Whitlatch, 2007; Menne et al., 2008). Of note, we did not find any studies that met our criteria (including the exclusion of neural imaging findings) and examined the association between emotion regulation and decision-making in certain dementia subtypes (i.e., DLB and vascular dementia). This issue also stands to benefit from future research.

In the five studies identified, evidence on response-focused processes was limited to measures relating to emotional experience. In these studies, common emotional symptoms in dementia (e.g., apathy, loss of empathy) were each evaluated in a single study (Table 1). Furthermore, we found no evidence relating to the use of response-focused emotion regulation strategies. These strategies can be associated with self-processes in older persons (English & John, 2013; Perach et al., 2020). For example, expressive suppression (a strategy that involves the inhibition of ongoing emotional behavior; Gross, 2015) has been associated with self-views relating to inauthenticity (English & John, 2013) and interpersonal standing (Perach et al., 2020) in older persons. Because decision-making in dementia can potentially be construed via core aspects of self-regulation, including emotion regulation (Farina et al., 2020), the above (lack of) findings highlight the need for future research regarding the associations between response-focused emotion regulation strategies and decision-making in dementia. In addition, whereas response-focused emotion regulation can potentially affect decision-making in emotionally affected dementia populations (Kligyte et al., 2013), evidence to date on the relationship between response-focused emotion regulation and decision-making in dementia is largely derived from research designs that do not inform temporality. Accordingly, studies that directly test the hypothesis that response-focused emotion regulation affects everyday decision-making in these populations are needed.

### Limitations and future directions

This review focused on emotion regulation as one factor that can potentially elucidate psychological processes that originate within the person with dementia and affect decision-making perceptions and behaviors and well-being outcomes (Farina et al., 2020). Based on the principles of scoping review methodology (Arksey & O'Malley, 2005), our methodology included a tailored, comprehensive search strategy, defined criteria, and narrative evidence synthesis. Specifically, we used a replicable search strategy that involved a thorough examination of different branches of the literature relating to our key constructs based on numerous recent reviews including five that used systematic electronic database searches. Nonetheless, it is possible that we did not identify all relevant studies (see Supplementary Appendix 2 for excluded studies). Whereas systematic review methodology is highly appropriate for questions “addressing the feasibility, appropriateness, meaningfulness or effectiveness of a certain treatment, or practice” (Munn et al., 2018; p. 3), this scoping review was not guided by a single question (such as the effects of a particular emotion regulation process/strategy on a particular type of everyday decision-making).

Further research is needed to establish causality between emotion regulation and everyday decision-making in dementia. Complementing the findings of this review, our research group is set to empirically examine these associations as a part of wider longitudinal investigation, that is, DETERMinants of quality of life, care and costs, and consequences of INequalities in people with Dementia and their carers (DETERMIND), including into the interconnections between core aspects of self-regulation, decision-making, and well-being in dementia (Farina et al., 2020).

The current investigation focused on emotion regulation as one core aspect of self-regulation (Williams et al., 2018), a construct that could potentially affect decision-making (Hershfield, 2011) and well-being in dementia. Indeed, research has already demonstrated that decision-making can potentially improve the well-being of persons with dementia. For example, qualitative studies have highlighted the importance of constructs that are conceptually related to decision-making (e.g., autonomy and goal pursuit) to quality of life in persons with dementia (O'Rourke et al., 2015). In addition, findings obtained from residents in care settings support the association between decision-making and improved well-being (e.g., Haslam et al., 2014; Langer & Rodin, 1976). For example, in a classic study, nursing home residents who were (vs. were not) encouraged to think about their autonomous decision-making and its meanings and given the opportunity to make decisions showed increased happiness and activity participation (Langer & Rodin, 1976). Thus, the current study serves to promote research on the associations between emotion regulation and decision-making to better understand well-being outcomes in dementia. Indeed, reflecting these gaps, our work in DETERMIND (Farina et al., 2020) is exploring the understudied associations of everyday decision-making, different emotion regulation strategies (e.g., positive reframing and venting; Carver, 1997), and well-being.

To support the development of a theoretical model of well-being in dementia that incorporates multiple core aspects of self-regulation (i.e., emotion regulation, cognition, and self-reflection; Williams et al., 2018) and decision-making (Farina et al., 2020), the DETERMIND study will examine also the associations between cognition or self-reflection factors and decision-making in dementia. Indeed, cognition affects decision-making (Ernst & Paulus, 2005) and qualitative associations between self-esteem and decision-making (Fetherstonhaugh et al., 2013) have been found. In addition, cognitive status has been positively associated with “living well” indices in dementia (Martyr et al., 2018) as have personality factors such as self-esteem, optimism, and self-efficacy (Lamont et al., 2019). Thus, investigating the possibility that core aspects of self-regulation interact to affect decision-making and subsequent well-being in dementia is an important direction for our research (Farina et al., 2020).

## Conclusions

This review serves to clarify the concept of everyday decision-making in dementia and to map the current state of evidence on its associations with emotion regulation. We examined the quantitative associations between antecedent- and response- focused emotion regulations and everyday decision-making in dementia. We found that, based on two studies, antecedent-focused processes that involve emotion recognition or arousal may be associated with moral decision-making in FTLD. In addition, we found that evidence concerning the association between response-focused emotion regulation and decision-making was derived from studies utilizing various methodologies and different dementia populations. We found no studies relating to the associations between decision-making in dementia and several antecedent-focused emotion regulation processes and response-focused strategies. In conclusion, this review provides initial support to the notion that emotion regulation may be an important predictor of decision-making in dementia and outlines the gaps in the literature to set a research agenda for promoting our understanding of how individual resources could improve well-being in dementia.

## Supplemental Material

sj-pdf-1-dem-10.1177_1471301220971630 – Supplemental Material for Emotion regulation and decision-making in persons with dementia: A scoping review Click here for additional data file. Supplemental Material, sj-pdf-1-dem-10.1177_1471301220971630 for Emotion regulation and decision-making in persons with dementia: A scoping review by Rotem Perach, Jennifer Rusted, Peter R. Harris and Eleanor Miles in Dementia
