# Supplementary material for: Emotion regulation and decision-making in persons with dementia: A scoping review
Source: Dementia (London). 2020 Nov 23;20(5):1832–54. doi: 10.1177/1471301220971630 (PMC8216314; doi:10.1177/1471301220971630)
Supplement: sj-pdf-1-dem-10.1177_1471301220971630 – Supplemental Material for Emotion regulation and decision-making in persons with dementia: A scoping review [file sj-pdf-1-dem-10.1177_1471301220971630.pdf]

Supplemental Material[1]

Appendix 1. Decision-making tasks' terminology and decision-making measures that met our criteria

1. Labels of decision-making tasks reviewed via search strategy

Balloon analogue risk task

Beads task

Bechara's card test

Binary choice task

Cambridge gambling/gamble task (Rogers et al., 1999)

In this computerized decision-making under risk task (also labelled "Roger's decision-making task"), participants are presented with 10 (red or blue) boxes and asked to bet a sum of their choice on whether a token was hidden in a red or blue box. The colour ratio of the 10 boxes, which informs winning probabilities, changes per round.

Chaining task

Choice task

Choice titration task

Columbia card task

Complex dynamic control

Deal or no-deal

Dynamic decision making

Dynamic foraging task

Dynamometer

Effort-based decision task

[1] A list of the complete citations of the papers cited in this document is available upon request.

Face decision task

Framing task/paradigm

Game of dice task (Brand et al., 2005)

In this computerized decision-making under risk task, participants aim to maximize financial gain. A virtual dice is thrown on each round, and participants need to decide which number or combination of numbers may include the number thrown. Information relating to the winning probabilities of the different alternatives (two advantageous, two disadvantageous) is presented to participants.

Information sampling task

Intertemporal choice

Intertemporal choice task

Investment task

Iowa gambling task (Bechara, Damasio, Damasio, & Anderson, 1994)

In the original version of this decision-making under uncertainty task, participants receive an initial sum of fake money and are instructed to try and maximize long-term financial gains by selecting one of four deck of cards on each round. Two decks bring higher immediate reward and higher penalty (and are therefore disadvantageous) and the other two decks bring lower immediate reward and lower penalty (and are therefore advantageous).

Kirby delayed discounting questionnaire

Monetary incentive delay

Monetary reward/loss risk-taking task

Moral decision-making

Moral dilemma paradigm

Moral judgement interview/task

Moving-dots task

Perceptual inference task

Pixel task

Probabilistic classification/category learning task (Shohamy, Myers, Onlaor, & Gluck, 2004)

> In this computerized decision-making under uncertainty task (also labelled the "weather prediction" task), participants are asked to predict the weather (sun or rain) based on one to three tarot cards presented on each round. Each card is associated with a fixed probability per weather outcome. No probability information is presented to participants.

Probabilistic reversal learning

Probabilistic reward and punishment learning task

Probabilistic reward task

Probabilistic selection task

Probability-associated gambling task (Zamarian, Sinz, Bonatti, Gamboz, & Delazer, 2008)

> In this computerized decision-making under risk task, participants aim to win as much money as possible by deciding on each round whether to take a small win/loss sum or gamble a larger sum. The chance of winning is presented visually as the ratio between red and blue cubes and changes per round.

Procedural learning transitive inference task

Risk task

Risk Perception in Investment Decisions task

Roger's decision-making task

Slot machine game

Sugar production task (Berry & Broadbent, 1984)

In this computerized decision-making under uncertainty task (also labelled a dynamic decision-making task), participants act as a sugar factory manager and need to maintain production output by assigning a number of workers for employment per trial. The results of each trial are shaped by one's previous decisions and pre-determined task probabilities. Participants receive cumulative feedback on their decisions.

Temporal discounting task

The giving game

Valuation rating task

Vancouver gambling task

Ultimatum game

2. Measures of decision-making that met our criteria

Decision-making Involvement Scale (Menne, Tucke, Whitlatch, & Feinberg, 2008)

Assessment for Capacity of Everyday Decision-making (Lai et al., 2008)

Appendix 2. List of excluded quantitative studies

Study*	Excluded based on inclusion criterion	Excluded based on exclusion criterion
	Number	
Al-Khaled, Heldmann, Bolstorff, Hagenah, & Münte, 2015	1	
Angioletti, Siri, Meucci, Pezzoli, & Balconi, 2019	1	
Baez et al., 2014	2	
Baez et al., 2016	2	
Biella et al., 2019	2	
Boller et al., 2014	1	
Cohen et al., 2016	2	
Djamshidian, O'Sullivan, Lees, & Averbeck, 2012	1	1
Euteneuer et al., 2009	1	
Evens et al., 2015	1	
Funkiewiez, Bertoux, de Souza, Lévy, & Dubois, 2012	2, 3	
Gleichgerrcht, Torralva, Roca, & Manes, 2010	2	
Grossman et al., 2010		1
Hanby et al., 2014	1	
Ibarretxe-Bilbao et al., 2009	1	
Jacus, Gély-Nargeot, & Bayard, 2018	2	
Kloeters, Bertoux, O'Callaghan, Hodges, & Hornberger, 2013	5	
Kobayakawa, Koyama, Mimura, & Kawamura, 2008	1	
Kobayakawa, Tsuruya, & Kawamura, 2010	1	
Martínez-Horta, Pagonabarraga, de Bobadilla, García-Sanchez, &	1	

Kulisevsky, 2013		
Martínez-Horta et al., 2014	1	
Melloni et al., 2016	2	
Mendez, Anderson, & Shapira, 2005		1
Mendez & Shapira, 2009		1
Milenkova et al., 2011	1	
Mimura, Oeda, & Kawamura, 2006	1	
O’Callaghan et al., 2015	5	
Oyama et al., 2011	1	
Pagonabarraga et al., 2007	1	
Perretta, Pari, & Beninger, 2005	1	
Poletti et al., 2010	1	
Poletti et al., 2011	1	
Poletti et al., 2012	1	
Rahman, Sahakian, Hodges, Rogers, & Robbins, 1999	1	
Rosen, Brand, Polzer, Ebersbach, & Kalbe, 2013	1	
Rosen, Rott, Ebersbach, & Kalbe, 2015	1	
Sáez-Francàs et al., 2014	1	
Simioni, Dagher, & Fellows, 2012	1	
Sinz, Zamarian, Benke, Wenning, & Delazer, 2008	2	
Sturm et al., 2017	5	
Sturm et al., 2015	2	
Perry, Sturm, Wood, Miller, & Kramer, 2015	2	
Werner et al., 2007	2	
Xi et al., 2015	1	
Yerstein, Carr, Jimenez, & Mendez, 2020	5	
Zamarian, Benke, Brand, Djamshidian, & Delazer, 2015	2	

Zamarian, Benke, Buchler, Wenter, & Delazer, 2010	2

Notes. The list presented in this table presents a selection of excluded studies that met some (but not all) of our criteria. Inclusion criterion 1 = Participants (full sample or a distinct group) had (any type of) dementia based on a diagnosis by a clinical neurologist or were described as meeting published clinical diagnostic criteria; 2 = Peer-reviewed journal article that reported new quantitative data in English on the association between everyday decision-making and emotion regulation in persons with dementia; 3 = We operationalized everyday decision-making as both tasks identified as assessing decision-making (e.g., under uncertainty, under risk, moral) and measures labelled as decision-making assessments; 5 = While decision-making in dementia may involve others (e.g., carers), our focus was on the person with dementia's perspective on individual factors that may influence everyday decision-making (Farina et al., 2020). Therefore, we only included studies in which the person with dementia was the source of evidence (De Boer et al., 2007); Exclusion criterion 1 = Emotion was not assessed independently of decision-making (e.g., moral dilemmas were labelled as 'emotional' (Mendez & Shapira, 2009).
